# Hyperhomocysteinaemia Promotes Doxorubicin-Induced Cardiotoxicity in Mice

**DOI:** 10.3390/ph16091212

**Published:** 2023-08-28

**Authors:** Rui Fan, Yao Wang, Jinjin Zhang, Xiangbo An, Shuang Liu, Jie Bai, Jiatian Li, Qiuyue Lin, Yunpeng Xie, Jiawei Liao, Yunlong Xia

**Affiliations:** 1 Institute of Cardiovascular Diseases, First Affiliated Hospital of Dalian Medical University, Dalian 116011, China; 2 Department of Interventional Therapy, First Affiliated Hospital of Dalian Medical University, Dalian 116011, China; 3 College of Basic Medical Sciences, Dalian Medical University, Dalian 116004, China; 4 Department of Nutrition and Food Hygiene, School of Public Health, Dalian Medical University, Dalian 116004, China

**Keywords:** hyperhomocysteinaemia, homocysteine, methionine, doxorubicin, cardiotoxicity, folic acid

## Abstract

Doxorubicin, a widely used chemotherapeutic drug in clinical oncology, causes a series of cardiac side effects referred to as doxorubicin-induced cardiotoxicity. Hyperhomocysteinaemia is an independent risk factor for multiple cardiovascular diseases. However, whether hyperhomocysteinaemia contributes to doxorubicin-induced cardiotoxicity is currently unknown. In this study, we explored the pathogenic effects of hyperhomocysteinaemia induced by dietary methionine supplementation (2% wt/wt in rodent chow) in a mouse model of doxorubicin-induced cardiotoxicity. Our data showed that methionine supplementation doubled serum homocysteine levels, inducing mild hyperhomocysteinaemia. Doxorubicin at a cumulative dosage of 25 mg/kg body weight led to significant weight loss and severe cardiac dysfunction, which were further exacerbated by methionine-induced mild hyperhomocysteinaemia. Doxorubicin-induced cardiac atrophy, cytoplasmic vacuolisation, myofibrillar disarray and loss, as well as cardiac fibrosis, were also exacerbated by methionine-induced mild hyperhomocysteinaemia. Additional folic acid supplementation (0.006% wt/wt) prevented methionine-induced hyperhomocysteinaemia and inhibited hyperhomocysteinaemia-aggravated cardiac dysfunction and cardiomyopathy. In particular, hyperhomocysteinaemia increased both serum and cardiac oxidative stress, which could all be inhibited by folic acid supplementation. Therefore, we demonstrated for the first time that hyperhomocysteinaemia could exacerbate doxorubicin-induced cardiotoxicity in mice, and the pathogenic effects of hyperhomocysteinaemia might at least partially correlate with increased oxidative stress and could be prevented by folic acid supplementation. Our study provides preliminary experimental evidence for the assessment of hyperhomocysteinaemia as a potential risk factor for chemotherapy-induced cardiotoxicity in cancer patients.

## 1. Introduction

Doxorubicin, or Adriamycin, is an anthracycline antibiotic isolated from the bacteria *Streptomyces peucetius var. caesius* in 1969 [1]. Ever since its first introduction into clinical oncology in the 1970s [2,3], doxorubicin has been widely used as a chemotherapeutic drug for solid tumours (e.g., breast cancer, pediatric osteosarcoma and hepatoblastoma, etc.) and hematologic malignancies (e.g., acute lymphoblastic leukaemia, Hodgkin/non-Hodgkin lymphoma, etc.) [4]. Although doxorubicin has powerful anti-cancer potential, it also causes severe side effects that restrict its clinical application. One of its most serious side effects is a series of dose-dependent, cumulative and progressive cardiac injuries referred to as doxorubicin-induced cardiotoxicity, which can occur as early as the first dosage or be delayed until decades after the last administration [5,6,7]. Ranging from early-onset electrocardiography abnormalities and asymptomatic cardiac dysfunction to chronic cardiomyopathy, doxorubicin-induced cardiotoxicity ultimately leads to congestive heart failure and even cardiac death, therefore significantly affecting the life quality and expectancy of the cancer survivors [5,6,7]. Previously, approximately 3–5% of cancer patients are estimated to develop heart failure when the accumulative doxorubicin dose reaches 400 mg/m^2^, whereas the incidence of heart failure dramatically increases to 7–26% and 18–48% when the accumulative doxorubicin doses reach 550 mg/m^2^ and 700 mg/m^2^, respectively [8]. However, recent guideline from the American Society of Clinical Oncology indicates that the reported incidence of cardiac dysfunction might be underestimated due to limitations of heart failure reporting on clinical trials and lack of long-term follow-up [9]. According to updated statistics, about 9% of patients develop cardiac dysfunction at a cumulative doxorubicin dose of 250 mg/m^2^, and the incidence rates jump to 18% and 38% at cumulative doxorubicin doses of 350 mg/m^2^ and 450 mg/m^2^, respectively [9]. In light of the low threshold for doxorubicin-induced cardiotoxicity, there is an urgent need to elucidate the risk factors and underlying mechanisms for better prevention and treatment of the disease.

Homocysteine is a sulfur-containing non-essential amino acid [10,11]. As an intermediate product of methionine metabolism, homocysteine is formed by a three-step sequential cascade mediated by S-adenosyl-L-methionine synthase, methyltransferase and S-adenosyl-L-homocysteine hydrolase, a process known as the transmethylation of the methionine [10,11]. Homocysteine can then either accept a methyl group via folate/vitamin B12-dependent as well as folate/vitamin B12-independent re-methylation to transform back into methionine or metabolised to form cysteine via cystathionine β-synthase and cystathionine γ-lyase-dependent trans-sulfuration pathway [10,11]. 

Generally, all tissues can produce homocysteine. However, the circulating homocysteine concentration is normally maintained below 10 μmol/L [10,11]. Nutritional factors (e.g., excessive methionine intake, dietary vitamin B12 or folate shortage) or genetic defects disrupting the transmethylation, re-methylation or trans-sulfuration pathway (e.g., genetic polymorphisms (commonly C677T and A1298C) in methylene tetrahydrofolate reductase or inherited deficiency in cystathionine β-synthase) can impair homocysteine homeostasis, leading to an elevation of circulating homocysteine concentration that subsequently induces devastating cardiovascular consequences, such as coronary heart diseases, stroke, and aneurysm [10,11]. In addition, an increase in circulating homocysteine can also be induced by some medication side effects (e.g., methotrexate, theophylline, phenytoin, and cyclosporine) or chronic diseases (e.g., end-stage renal disease, severe hepatic dysfunction, diabetes mellitus, and hypothyroidism) [12]. 

For the past three decades, more and more evidence has indicated that hyperhomocysteinaemia, defined as a circulating homocysteine concentration ≥15 μmol/L, is an independent risk factor for cardiovascular diseases (especially vascular diseases), although the pathogenesis is not fully elucidated [13,14]. However, whether hyperhomocysteinaemia could contribute to doxorubicin-induced cardiotoxicity is currently unknown. In this study, we explored the pathogenic effects of hyperhomocysteinaemia in a mouse model of doxorubicin-induced cardiotoxicity induced by doxorubicin injection. We also explored whether folic acid supplementation could rescue hyperhomocysteinaemia and inhibit doxorubicin-induced cardiotoxicity in the current hyperhomocysteinemic model.

## 2. Results

### 2.1. Methionine-Induced Hyperhomocysteinaemia Exacerbates Doxorubicin-Induced Cardiac Dysfunction

**Figure 1:**
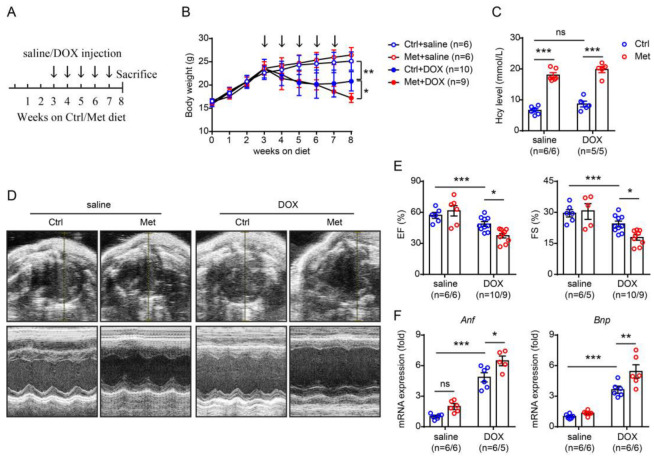
Methionine (Met)-induced hyperhomocysteinaemia exacerbates doxorubicin-induced cardiac dysfunction. (**A**) Schematic illustration of the experimental design. (**B**) Body weight during the experimental process. The arrow indicates a single doxorubicin (DOX) injection. (**C**) Serum homocysteine (Hcy) concentration at the end of the experiment. (**D**) Representative M-mode echocardiography at the end of the experiment. (**E**) Calculation of ejection fraction (EF%, left) and fractional shortening (FS%, right). (**F**) Quantitative real-time PCR analysis of cardiac *Anf* (left) and *Bnp* (right) expression. n = 6–10 per group. *: *p* < 0.05; **: *p* < 0.01; ***: *p* < 0.001; ns—not significant.

To explore the effect of homocysteine on doxorubicin-induced cardiotoxicity, mice were fed with a methionine-supplemented (2% methionine wt/wt) diet to induce hyperhomocysteinaemia, and those who received normal rodent chow diet feeding were used as controls. After three weeks of the diet feeding, mice were subjected to five consecutive injections of doxorubicin (5 mg/kg body weight per week) or the same amount of saline (Figure 1A). During the experimental period, the body weight of the mice was recorded once per week. As shown in Figure 1B, doxorubicin injection induced progressive weight loss, which was exacerbated by methionine-supplemented diet feeding (Figure 1B). At the end-point of the experiment, circulating homocysteine levels were compared between the methionine-supplemented and the control diet groups. We found that doxorubicin injections did not elicit a remarkable impact on circulating homocysteine concentrations. However, methionine-supplemented diet feeding doubled circulating homocysteine levels as compared with those received control diet feeding (Figure 1C). Doxorubicin, at a cumulative dosage of 25 mg/kg body weight, induced severe cardiac dysfunction, as indicated by a significant decrease in ejection fraction (EF) and fractional shortening (FS) in echocardiography (Figure 1D,E) and a significant increase in cardiac atrial natriuretic factor (*Anf*) and B-type natriuretic peptide (*Bnp*) expression by quantitative real-time PCR (Figure 1F). Methionine-induced hyperhomocysteinaemia caused a further reduction of EF and FS (Figure 1D,E) as well as a further increase in *Anf* and *Bnp* levels (Figure 1F), suggesting that methionine-induced hyperhomocysteinaemia exacerbated doxorubicin-induced cardiac dysfunction. 

### 2.2. Methionine-Induced Hyperhomocysteinaemia Exacerbates Doxorubicin-Induced Cardiomyopathy

**Figure 2:**
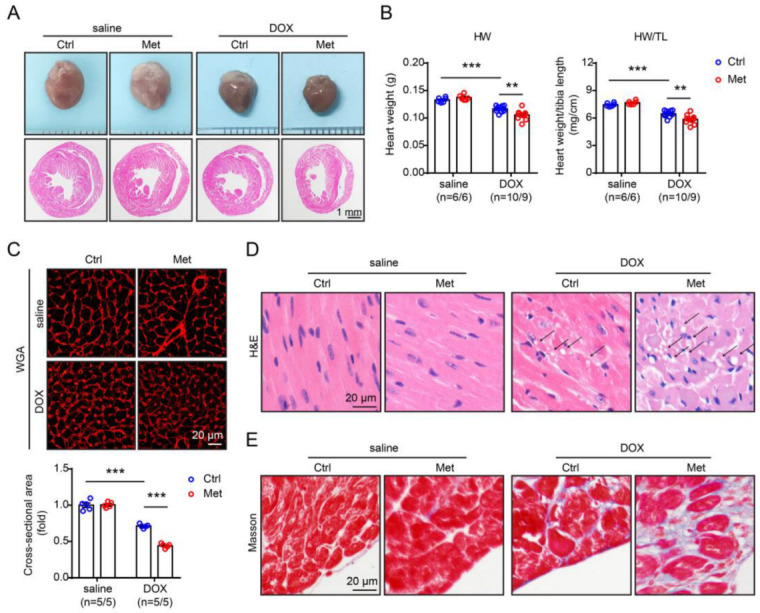
Methionine (Met)-induced hyperhomocysteinaemia exacerbates doxorubicin-induced cardiomyopathy. (**A**) Representative gross appearance (upper) and cross-sectional H&E staining (lower) of the heart. (**B**) Heart weight (left) and heart weight/tibial length ratio (right). (**C**) Representative WGA staining of the heart sections (upper) and quantitation of myocyte cross-sectional areas (lower). (**D**) Representative H&E staining of the heart sections. The arrow indicates cytoplasmic vacuolisation. (**E**) Representative Masson staining of the heart sections. n = 6–10 per group. **: *p* < 0.01; ***: *p* < 0.001.

Doxorubicin-induced cardiomyopathies in animal models are typically characterised by cardiac myocyte atrophy, cytoplasmic vacuolisation, as well as myofibrillar disarray and loss [15]. Inflammatory cells can be absent in doxorubicin-induced cardiomyopathy, but fibrosis usually develops in the late stage [15]. Our data showed that in the methionine-induced hyperhomocysteinaemia group, doxorubicin-induced cardiac atrophy was more prominent, as shown by a further reduction of heart size (Figure 2A) as well as a further decrease in heart weight and heart weight/tibial length ratio, suggesting that methionine-induced hyperhomocysteinaemia promoted doxorubicin-induced cardiac atrophy (Figure 2B). Exacerbation of doxorubicin-induced cardiac atrophy by methionine-induced hyperhomocysteinaemia was also confirmed by cardiomyocyte dihydroethidium (DHE) staining, which showed a further decrease in mean cardiomyocyte cross-sectional areas in the methionine-supplemented diet group, compared with those of the control diet group (Figure 2C). Moreover, H&E staining showed that doxorubicin-induced cytoplasmic vacuolisation, as well as myofibrillar disarray and loss, was exacerbated in the methionine-supplemented diet group (Figure 2D), while Masson staining showed that doxorubicin-induced cardiac fibrosis was also exacerbated in the methionine-supplemented diet group (Figure 2E).

### 2.3. Folic Acid Supplementation Inhibits Methionine-Induced Hyperhomocysteinaemia and Its-Associated Cardiac Dysfunction

**Figure 3:**
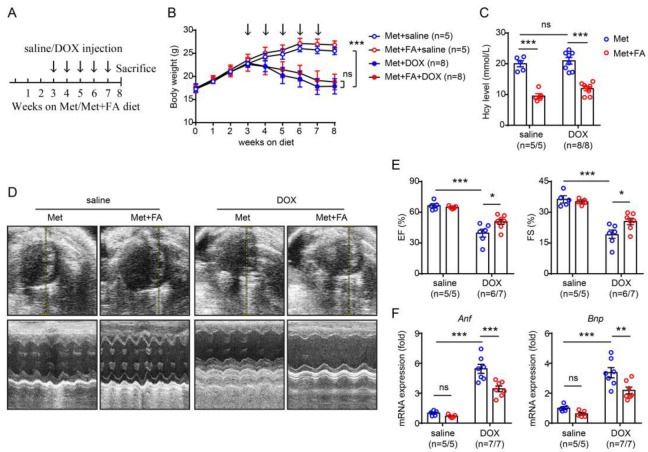
Folic acid (FA) supplementation inhibits methionine-induced hyperhomocysteinaemia and hyperhomocysteinaemia-aggravated cardiac dysfunction. (**A**) Schematic illustration of the experimental design. (**B**) Body weight during the experimental process. The arrow indicates a single doxorubicin (DOX) injection. (**C**) Serum homocysteine (Hcy) concentration at the end of the experiment. (**D**) Representative M-mode echocardiography at the end of the experiment. (**E**) Calculation of ejection fraction (EF%, left) and fractional shortening (FS%, right). (**F**) Quantitative real-time PCR analysis of cardiac *Anf* (left) and *Bnp* (right) expression. n = 5–8 per group. *: *p* < 0.05; **: *p* < 0.01; ***: *p* < 0.001; ns—not significant.

We next treated methionine-fed mice with an additional 0.006% wt/wt folic acid in their chow to test whether folic acid could inhibit hyperhomocysteinaemia-exacerbated cardiotoxicity (Figure 3A). As shown in Figure 3B, folic acid supplementation only had a mild impact on doxorubicin-induced weight loss in the methionine-fed mice during the doxorubicin injection process (Figure 3B). However, at the end-point of the experiment, folic acid supplementation almost fully reversed methionine-induced hyperhomocysteinaemia (Figure 3C). By inhibiting hyperhomocysteinaemia, folic acid supplementation remarkably improved doxorubicin-induced cardiac dysfunction, as demonstrated by a significant increase in EF and FS (Figure 3D,E) and a significant decrease in *Anf* and *Bnp* expression (Figure 3F) in the methionine-fed mice subjected to doxorubicin injections. 

### 2.4. Folic Acid Supplementation Reduces Hyperhomocysteinaemia-Aggravated Cardiomyopathy

**Figure 4:**
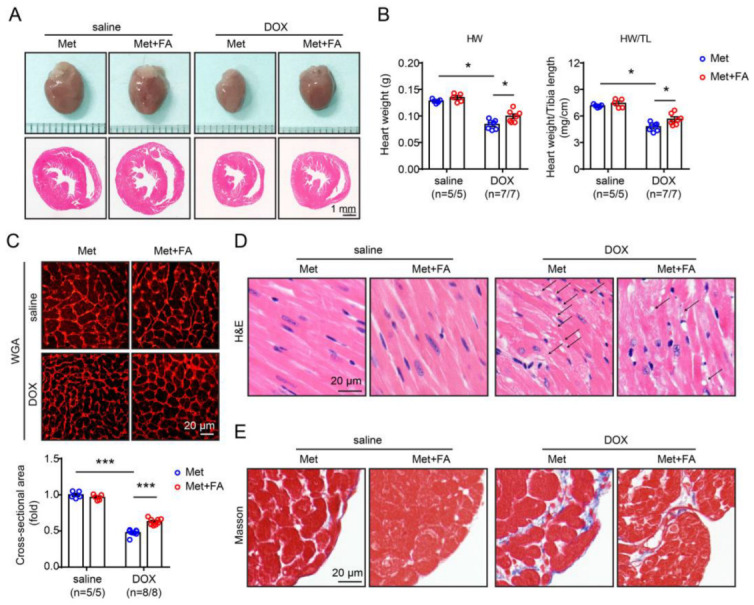
Folic acid (FA) supplementation inhibits hyperhomocysteinaemia-aggravated cardiomyopathy. (**A**) Representative gross appearance (upper) and cross-sectional H&E staining (lower) of the heart. (**B**) Heart weight (left) and heart weight/tibial length ratio (right). (**C**) Representative WGA staining of the heart sections (upper) and quantitation of myocyte cross-sectional areas (lower). (**D**) Representative H&E staining of the heart sections. The arrow indicates cytoplasmic vacuolisation. (**E**) Representative Masson staining of the heart sections. n = 5–8 per group. *: *p* < 0.05; ***: *p* < 0.001.

The impact of folic acid supplementation on doxorubicin-induced cardiomyopathy was also evaluated. We found that folic acid supplementation significantly reduced doxorubicin-induced cardiac atrophy in the methionine-fed mice, as shown by a relative increase in heart size (Figure 4A) as well as a relative increase in heart net weight and heart weight/tibial length ratio (Figure 4B) in the folic acid-supplemented mice receiving doxorubicin injections. Improvement of doxorubicin-induced cardiac atrophy in the folic acid-supplemented mice was further confirmed by calculating cardiomyocyte cross-sectional areas in WGA staining (Figure 4C). Folic acid supplementation also improved doxorubicin-induced cytoplasmic vacuolisation, myofibrillar disarray and loss shown in H&E staining (Figure 4D), as well as cardiac fibrosis shown in Masson staining (Figure 4E) in the methionine-fed group. 

### 2.5. Increased Oxidative Stress Might Contribute to Hyperhomocysteinaemia-Aggravated Cardiomyopathy

**Figure 5:**
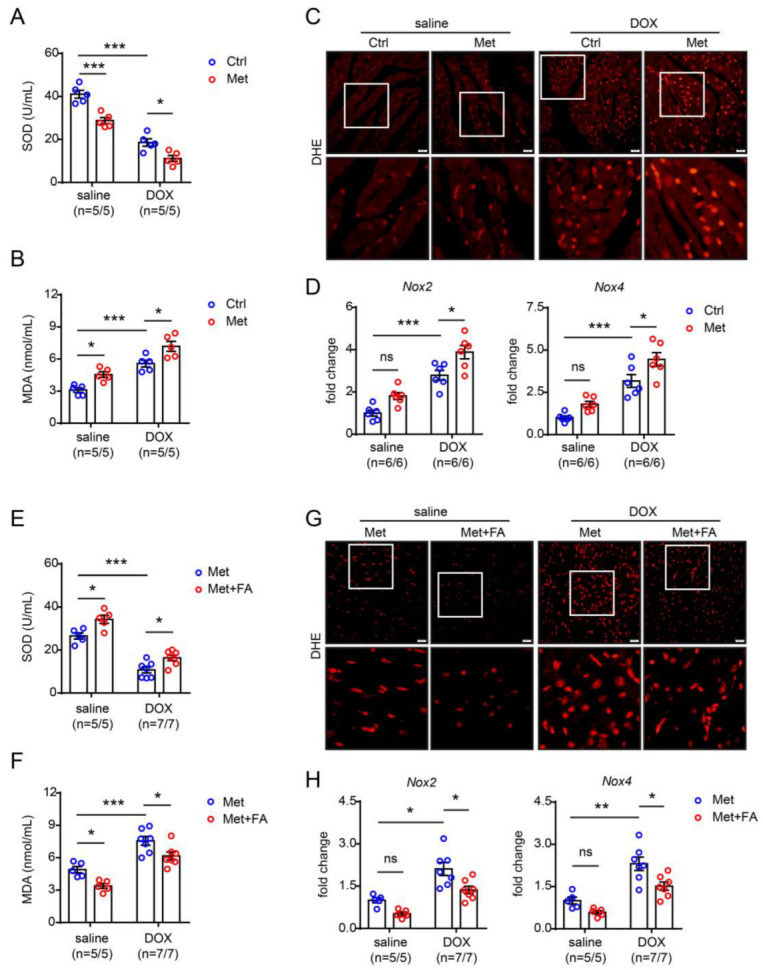
Increased oxidative stress might contribute to hyperhomocysteinaemia-aggravated cardiomyopathy. (**A**) Serum superoxide dismutase (SOD) content in methionine (Met)-fed hyperhomocysteinemic mice. (**B**) Serum malondialdehyde (MDA) content in methionine (Met)-fed hyperhomocysteinemic mice. (**C**) Representative dihydroethidium (DHE) staining of the heart sections in methionine (Met)-fed hyperhomocysteinemic mice, scale bar = 20 μm. (**D**) Quantitative real-time PCR analysis of cardiac *Nox2* (left) and *Nox4* (right) expression in methionine (Met)-fed hyperhomocysteinemic mice. (**E**) Serum superoxide dismutase (SOD) content in methionine (Met)-fed mice with folic acid (FA) supplementation. (**F**) Serum malondialdehyde (MDA) content in methionine (Met)-fed mice with folic acid (FA) supplementation. (**G**) Representative DHE staining of the heart sections in methionine (Met)-fed mice with folic acid (FA) supplementation, scale bar = 20 μm. (**H**) Quantitative real-time PCR analysis of cardiac *Nox2* (left) and *Nox4* (right) expression in methionine (Met)-fed mice with folic acid (FA) supplementation. n = 5–7 per group. *: *p* < 0.05; **: *p* < 0.01; ***: *p* < 0.001; ns—not significant.

Oxidative stress is caused by an imbalance between antioxidants and reactive oxygen species (ROS) [16]. Usually, the activities of the antioxidants, including superoxide dismutase, catalase, and reduced glutathione, as well as lipid peroxidation levels indicated by malondialdehyde content, are used to evaluate the level of oxidative stress in the serum [17,18], while the level of oxidative stress in the cardiac tissue is evaluated by cardiac DHE fluorescent intensity and ROS-generating enzyme NADPH oxidase (NOX) expression [19,20]. Here, we showed that doxorubicin significantly decreased serum superoxide dismutase but increased malondialdehyde content, which was further exacerbated by methionine-induced hyperhomocysteinaemia (Figure 5A,B). Furthermore, doxorubicin significantly increased cardiac DHE fluorescent intensity as well as *Nox2* and *Nox4* gene expression, which was also further exacerbated by methionine-induced hyperhomocysteinaemia (Figure 5C,D). Folic acid supplementation, by inhibiting the increase in circulating homocysteine concentration, protected against increased serum (Figure 5E,F) and cardiac oxidative stress (Figure 5G,H). Notably, hyperhomocysteinaemia itself increased serum and cardiac oxidative stress, which could also be inhibited by folic acid, even without doxorubicin treatment (Figure 5).

## 3. Discussion

In this study, we demonstrated for the first time that: (1) Methionine-induced hyperhomocysteinaemia exacerbated doxorubicin-induced cardiac dysfunction and cardiomyopathy in mice; (2) folic acid inhibited methionine-induced hyperhomocysteinaemia and associated cardiotoxic effects; and (3) hyperhomocysteinaemia decreased circulating antioxidants and increased cardiac ROS levels, which could be inhibited by folic acid supplementation. 

Homocysteine, as described above, is an intermediate product of methionine metabolism. Therefore, is closely associated with methionine homeostasis [10,11]. Being an essential sulfur-containing amino acid, methionine is normally contained in dietary protein, and the major sources of methionine include eggs, fish, meat, and cereal grains [10,11]. For rodents, the average content of methionine in normal chow diets is 5–7 g/kg [21]. Increased daily methionine intake by dietary methionine supplementation has been used to induce experimental hyperhomocysteinaemia. Notably, there is evidence showing that 2.7% methionine supplementation to the normal rodent chow diet reduces food intake and causes severe growth suppression, thymus atrophy, changes in plasma biochemical parameters, and abnormal organ histology in rats, indicating broad toxicity of high dietary methionine supplementation [22]. In comparison, 0.9% methionine supplementation leads to mild growth suppression and a minor change in plasma biochemical parameters, whereas 0.3% supplementation hardly results in any adverse effect [22]. More importantly, methionine supplementation between 0.3% and 0.9% might produce potential metabolic benefits via the modulation of various metabolic factors and epigenetic regulations [23,24,25,26]. Therefore, supplementation of methionine between 0.3% and 0.9% might be considered a relatively safe or even healthy range, at least in rodents [24]. In the current study, we used a normal rodent chow diet with 2% methionine supplementation to induce hyperhomocysteinaemia in mice. The methionine content in this diet is significantly higher than the safe range (0.3–0.9%) but lower than the toxic level (2.7%) and has been demonstrated to cause a mild-to-moderate increase in circulating homocysteine levels without gross observation of severe adverse effects in mice [27,28]. 

As a prevalent metabolic disorder, hyperhomocysteinaemia is well established as an independent risk factor for vascular diseases, such as coronary heart disease, stroke and aneurysm [10,11]. Recently, we have demonstrated that hyperhomocysteinaemia also promotes pathological cardiac hypertrophy in hypertension, and its pro-hypertrophic effect at least involves Calcineurin-NFAT signalling [29]. Apart from profound impacts on the cardiovascular system, hyperhomocysteinaemia is increasingly recognised as a potential risk factor as well as a biomarker for a variety of cancers, including breast cancer, leukaemia, and colorectal cancer, as summarised in a recent review [30,31]. Hyperhomocysteinaemia not only facilitates the onset and progression of cancers but also affects the development of cancer-related complications, such as venous and arterial thrombosis [31]. In fact, hyperhomocysteinaemia has been suggested as the most common factor correlated with the formation of venous thromboembolism in cancer patients, although the precise underlying mechanisms are not fully elucidated [32]. In addition to cancer-related complications, cancer therapy-associated complications are another important aspect that significantly affects the prognosis of cancer survivors. Of which, cancer therapy-associated cardiotoxicity, especially chemotherapy-induced cardiotoxicity, is a cause of cancer-related deaths second only to the cancer itself [33,34]. However, whether hyperhomocysteinaemia could be a contributory factor to chemotherapy-induced cardiotoxicity is still unknown. Here we provided the first experimental evidence that hyperhomocysteinaemia promoted doxorubicin-induced cardiac dysfunction and cardiomyopathy, indicating that cancer survivors with hyperhomocysteinaemia might have an increased risk of developing cardiotoxicity upon chemotherapy treatment compared to those without hyperhomocysteinaemia. Therefore, their circulating homocysteine concentration during and after cancer therapy should be closely monitored. 

Folate, or vitamin B9, an essential nutrient from the vitamin B group, is found naturally rich in fruits and green leafy vegetables [12,35]. Its derivative, tetrahydrofolate, is the central acceptor molecule in the one-carbon cycle, a process crucial for purine and pyrimidine biosynthesis [12]. In clinics, folate supplementation is routinely used to prevent and treat neural tube defects, megaloblastic anaemia, and cognitive decline [36,37]. As a cofactor for methylenetetrahydrofolate reductase, folate also contributes to homocysteine homeostasis via mediating the re-methylation of homocysteine into methionine and functions as the most important dietary determinant of circulating homocysteine levels [10,11]. In cancer patients, the rapid proliferation of tumour cells uses circulating folate for de novo purine synthesis. Therefore, it might induce folate shortage and subsequent hyperhomocysteinaemia, especially in the advanced stage of cancers [38,39]. In rodents, folic acid has been demonstrated to correct hyperhomocysteinaemia as well as hyperhomocysteinaemia-associated pathogenesis [40,41,42]. In humans, daily folic acid supplementation of 0.5–5.0 mg is estimated to produce an approximately 25% decrease in circulating homocysteine levels [12]. However, in contrast to experimental studies, lowering circulating homocysteine levels by folic acid supplementation is incapable of improving cardiovascular outcomes in some population-based studies [43,44,45,46,47]. In this study, we showed that folic acid could inhibit methionine-induced hyperhomocysteinaemia and, especially, its pathogenesis on doxorubicin-induced cardiotoxicity. Worth mentioning is that in a recent study, folic acid supplementation is demonstrated to protect against doxorubicin-induced cardiotoxicity by modulating endothelial nitric oxide synthase phosphorylation and nitric oxide production, which subsequently reduce oxidative stress and impairment of mitochondrial function [48]. In contrast to the current study, such a cardio-protective role of folic acid is observed without the context of hyperhomocysteinaemia. Although experimental evidence suggests a cardiac benefit of folic acid, whether folic acid could be used as a preventive and therapeutic option against doxorubicin-induced cardiotoxicity in cancer survivors still needs to be confirmed in further explorations. 

Although the underlying mechanisms for doxorubicin-induced cardiotoxicity are multifactorial and not fully elucidated, oxidative stress is one of the major well-established causes [49]. Most intracellular ROS are generated in the mitochondria, where ROS-producing enzymes transform doxorubicin to semiquinone [16]. Semiquinones can then react with oxygen to generate superoxide anions, which can be either transformed into hydrogen peroxide by superoxide dismutase or transferred to ROS or reactive nitrogen species [16]. In a subsequent iron-catalyzing Fenton reaction, superoxide anions and hydrogen peroxide might generate more active and toxic hydroxyl radicals, increasing intracellular oxidative stress [16]. Notably, cardiomyocytes contain 35–40% more mitochondria than other parenchymal cells [16]. The relatively high content of mitochondria in cardiomyocytes might partially explain the increased susceptibility of cardiomyocytes to doxorubicin-induced injury [50]. In the current study, we demonstrated that mild hyperhomocysteinaemia could exacerbate doxorubicin-induced increase in circulating oxidative stress levels, as indicated by a further decrease in superoxide dismutase and a further increase in the lipid peroxidation marker malondialdehyde in hyperhomocysteinemic mice; hyperhomocysteinaemia also exacerbated doxorubicin-induced increase in cardiac oxidative stress levels, as indicated by a significant increase in cardiac DHE fluorescent intensity as well as cardiac *Nox2* and *Nox4* expression. Interestingly, folic acid supplementation, by inhibiting the increase in serum homocysteine induced by methionine diet feeding, protected against the hyperhomocysteinaemia-aggravated increase in both circulating oxidative stress levels and cardiac oxidative stress injury. Therefore, hyperhomocysteinaemia-aggravated oxidative stress might at least partially contribute to hyperhomocysteinaemia’s pathogenic effects on doxorubicin-induced cardiotoxicity. 

## 4. Materials and Methods

### 4.1. Animals, Diets and Experimental Design

Male C56BL/6J wild-type mice aged 4–5 weeks were purchased from Beijing Vital River Laboratory (Beijing, China) and housed in individually ventilated cages with free access to diet and water. Diets used in this study include a normal rodent chow diet, a methionine diet (rodent chow diet supplemented with 2% (wt/wt) methionine) [27,28], and a methionine diet with additional folic acid (0.006% wt/wt) supplementation [51,52]. Cardiotoxicity was induced by five consecutive peritoneal injections of doxorubicin (MedChemExpress, Monmouth Junction, NJ, USA) at the dosage of 5 mg/kg body weight per week, as previously described [53,54]. 

Animal welfare and all experimental procedures were conducted according to the Guide for the Care and Use of Laboratory Animals (NIH Publication No. 85–23, revised 1996) and approved by the Animal Care and Use Committee of Dalian Medical University (approval number AEE-21079). 

### 4.2. Serum Biochemical Assay

Blood was collected by retro-orbital bleeding under ketamine-xylazine anaesthesia (ketamine: 0.2 g/kg body wt, xylazine: 0.01 g/kg body wt; intraperitoneal injection) and serum was obtained by centrifugation at 4000 rpm for 10 min in 4 °C. Serum homocysteine concentration was determined using a commercial Elisa kit (ml002038; Mlbio, Shanghai, China), according to the manufacturer’s guidance. Serum superoxide dismutase (SOD) (BC0175; Solarbio, Beijing, China) and malondialdehyde (MDA) (BC0025; Solarbio, Beijing, China) concentration was determined using commercial kits from Solarbio, according to the manufacturer’s guidance. 

### 4.3. Echocardiography

Mice were anaesthetized by 1.5% isoflurane inhalation. Transthoracic echocardiography was performed using Vevo 770 Micro-Ultrasound system (Visual Sonics, Toronto, ON, Canada) as previously described [55]. In brief, after the mouse heart rate stabilised at 400–500 beats per minute under isoflurane anaesthesia, parasternal long-axis images were obtained in B-mode, and the scan head was appropriately positioned to identify the maximum left ventricular length. Then the M-mode cursor was positioned perpendicular to the maximum left ventricular dimension in end-diastole and systole to obtain short-axis M-mode images, which were further used for measuring left ventricular wall thickness and chamber dimensions to calculate ejection fraction (EF) and fractional shortening (FS). Both EF and FS were averaged from three cardiac cycles.

### 4.4. Histopathology

Mice were sacrificed by CO_2_ inhalation and flushed by phosphate buffer saline through the left ventricle. The entire hearts were removed and weighed. After fixation in 4% paraformaldehyde solution (Life-iLab, Shanghai, China), hearts were embedded in paraffin and cross-sectioned at 5 μm thickness. Cardiac gross morphology and fibrosis were visualised by Hematoxylin and eosin (H&E) staining (G1120; Solarbio, Beijing, China) and Masson’s trichrome staining (G1340-7; Solarbio, Beijing, China), while cardiomyocyte size and reactive oxygen species (ROS) levels were visualised by rhodamine-labelled wheat germ agglutinin (WGA) (1.25 mg/mL; ZD0510, Vector Laboratory, Burlingame, CA, USA) staining and dihydroethidium (DHE) staining (1 µmol/L; Sigma-Aldrich, St. Louis, MO, USA) [56], respectively. Quantifications of WGA-stained cardiomyocyte cross-sectional areas and DHE fluorescent intensity were performed with Image J software (ImageJ1.51j8, NIH, United States).

### 4.5. Quantitative Real-Time PCR Analysis

RNA extraction and quantitative real-time PCR were performed as previously described [57]. In brief, total RNA was extracted using Tri-reagent (R1100; Solarbio, Beijing, China) and reverse-transcribed to first-strand complementary DNA with an RT Master Mix for qPCR (HY-K0501; MedChemExpress, Monmouth Junction, NJ, USA). Amplifications were performed in 35 cycles using the Applied Biosystems 7500 FAST real-time PCR system with SYBR Green qPCR reagents (AN19L919; Life-iLab, Shanghai, China). Each cycle consisted of heating denaturation for 30 s at 94 °C, annealing for 30 s at 56 °C, and extension for 30 s at 72 °C. All samples were quantitated using the comparative CT method for relative gene expression quantitation and normalised to β-actin levels. The primers used in this study are listed in Table 1. 

### 4.6. Statistical Analysis

All data were presented as Mean ± standard error of the mean (SEM). Statistical comparisons were evaluated by two-way ANOVA followed by Tukey’s test or the Mann–Whitney U-test for nonparametric data with GraphPad Prism software (Graphpad prism 7.00). A *p-value* < 0.05 was considered statistically significant.

## 5. Conclusions

In conclusion, the current study demonstrates for the first time that hyperhomocysteinaemia could exacerbate doxorubicin-induced cardiotoxicity in mice, and the pathogenic effects of hyperhomocysteinaemia might at least partially correlate with increased oxidative stress and could be prevented by folic acid supplementation. This study provides preliminary experimental evidence for the assessment of hyperhomocysteinaemia as a potential risk factor for chemotherapy-induced cardiotoxicity in cancer patients. Further studies are needed to define the underlying molecular mechanism of hyperhomocysteinaemia’s pathogenesis.

## Figures and Tables

**Table 1:** Primers used for quantitative real-time PCR analysis.

Gene	Forward Primer (5′–3′)	Reverse Primer (5′–3′)
*Anf*	CACAGATCTGATGGATTTCAAGA	CCTCATCTTCTACCGGCATC
*Bnp*	GAAGGTGCTGTCCCAGATGA	CCAGCAGCTGCATCTTGAAT
*Nox2*	ACCGGGTTTATGATATTCCACCT	GATTTCGACAGACTGGCAAGA
*Nox4*	CAGATGTTGGGGCTAGGATTG	GAGTGTTCGGCACATGGGTA
*β-actin*	ACTGCCGCATCCTCTTCCT	TCAACGTCACACTTCATGATGGA

## Data Availability

Data is contained within the article.

## Abbreviations

EF—ejection fraction; FS—fractional shortening; ROS—reactive oxygen species; WGA—wheat germ agglutinin; DHE—dihydroethidium; ANF—atrial natriuretic factor; BNP—B-type natriuretic peptide; NOX—NADPH oxidase.

## References

[1] Arcamone F., Cassinelli G., Fantini G., Grein A., Orezzi P., Pol C., Spalla C. (1969). Adriamycin, 14-hydroxydaunomycin, a new antitumor antibiotic from S. peucetius var. caesius. Biotechnol. Bioeng..

[2] Bonadonna G., Monfardini S., De Lena M., Fossati-Bellani F. (1969). Clinical evaluation of adriamycin, a new antitumour antibiotic. Br. Med. J..

[3] Bonadonna G., Monfardini S., De Lena M., Fossati-Bellani F., Beretta G. (1970). Phase I and preliminary phase II evaluation of adriamycin (NSC 123127). Cancer Res..

[4] van der Zanden S.Y., Qiao X., Neefjes J. (2021). New insights into the activities and toxicities of the old anticancer drug doxorubicin. FEBS J..

[5] Octavia Y., Tocchetti C.G., Gabrielson K.L., Janssens S., Crijns H.J., Moens A.L. (2012). Doxorubicin-induced cardiomyopathy: From molecular mechanisms to therapeutic strategies. J. Mol. Cell. Cardiol..

[6] Carvalho F.S., Burgeiro A., Garcia R., Moreno A.J., Carvalho R.A., Oliveira P.J. (2014). Doxorubicin-induced cardiotoxicity: From bioenergetic failure and cell death to cardiomyopathy. Med. Res. Rev..

[7] Rawat P.S., Jaiswal A., Khurana A., Bhatti J.S., Navik U. (2021). Doxorubicin-induced cardiotoxicity: An update on the molecular mechanism and novel therapeutic strategies for effective management. Biomed. Pharmacother..

[8] Zamorano J.L., Lancellotti P., Rodriguez Munoz D., Aboyans V., Asteggiano R., Galderisi M., Habib G., Lenihan D.J., Lip G.Y., Lyon A.R. (2017). 2016 ESC Position Paper on cancer treatments and cardiovascular toxicity developed under the auspices of the ESC Committee for Practice Guidelines: The Task Force for cancer treatments and cardiovascular toxicity of the European Society of Cardiology (ESC). Eur. J. Heart Fail..

[9] Armenian S.H., Lacchetti C., Barac A., Carver J., Constine L.S., Denduluri N., Dent S., Douglas P.S., Durand J.B., Ewer M. (2017). Prevention and Monitoring of Cardiac Dysfunction in Survivors of Adult Cancers: American Society of Clinical Oncology Clinical Practice Guideline. J. Clin. Oncol. Off. J. Am. Soc. Clin. Oncol..

[10] Kumar A., Palfrey H.A., Pathak R., Kadowitz P.J., Gettys T.W., Murthy S.N. (2017). The metabolism and significance of homocysteine in nutrition and health. Nutr. Metab..

[11] Zaric B.L., Obradovic M., Bajic V., Haidara M.A., Jovanovic M., Isenovic E.R. (2019). Homocysteine and Hyperhomocysteinaemia. Curr. Med. Chem..

[12] Kaye A.D., Jeha G.M., Pham A.D., Fuller M.C., Lerner Z.I., Sibley G.T., Cornett E.M., Urits I., Viswanath O., Kevil C.G. (2020). Folic Acid Supplementation in Patients with Elevated Homocysteine Levels. Adv. Ther..

[13] Clarke R., Daly L., Robinson K., Naughten E., Cahalane S., Fowler B., Graham I. (1991). Hyperhomocysteinemia: An independent risk factor for vascular disease. N. Engl. J. Med..

[14] Fu Y., Wang X., Kong W. (2018). Hyperhomocysteinaemia and vascular injury: Advances in mechanisms and drug targets. Br. J. Pharmacol..

[15] Podyacheva E.Y., Kushnareva E.A., Karpov A.A., Toropova Y.G. (2021). Analysis of Models of Doxorubicin-Induced Cardiomyopathy in Rats and Mice. A Modern View from the Perspective of the Pathophysiologist and the Clinician. Front. Pharmacol..

[16] Songbo M., Lang H., Xinyong C., Bin X., Ping Z., Liang S. (2019). Oxidative stress injury in doxorubicin-induced cardiotoxicity. Toxicol. Lett..

[17] He H., Luo Y., Qiao Y., Zhang Z., Yin D., Yao J., You J., He M. (2018). Curcumin attenuates doxorubicin-induced cardiotoxicity via suppressing oxidative stress and preventing mitochondrial dysfunction mediated by 14-3-3gamma. Food Funct..

[18] Yang B., Li H., Qiao Y., Zhou Q., Chen S., Yin D., He H., He M. (2019). Tetramethylpyrazine Attenuates the Endotheliotoxicity and the Mitochondrial Dysfunction by Doxorubicin via 14-3-3gamma/Bcl-2. Oxid. Med. Cell. Longev..

[19] Yan X., Zhang Y.L., Zhang L., Zou L.X., Chen C., Liu Y., Xia Y.L., Li H.H. (2019). Gallic Acid Suppresses Cardiac Hypertrophic Remodeling and Heart Failure. Mol. Nutr. Food Res..

[20] Bai J., Yin L., Yu W.J., Zhang Y.L., Lin Q.Y., Li H.H. (2022). Angiotensin II Induces Cardiac Edema and Hypertrophic Remodeling through Lymphatic-Dependent Mechanisms. Oxid. Med. Cell. Longev..

[21] Stone K.P., Wanders D., Orgeron M., Cortez C.C., Gettys T.W. (2014). Mechanisms of increased in vivo insulin sensitivity by dietary methionine restriction in mice. Diabetes.

[22] Chin K., Toue S., Kawamata Y., Watanabe A., Miwa T., Smriga M., Sakai R. (2015). A 4-week toxicity study of methionine in male rats. Int. J. Toxicol..

[23] Navik U., Sheth V.G., Kabeer S.W., Tikoo K. (2019). Dietary Supplementation of Methyl Donor l-Methionine Alters Epigenetic Modification in Type 2 Diabetes. Mol. Nutr. Food Res..

[24] Navik U., Sheth V.G., Khurana A., Jawalekar S.S., Allawadhi P., Gaddam R.R., Bhatti J.S., Tikoo K. (2021). Methionine as a double-edged sword in health and disease: Current perspective and future challenges. Ageing Res. Rev..

[25] Navik U., Sheth V.G., Sharma N., Tikoo K. (2022). L-Methionine supplementation attenuates high-fat fructose diet-induced non-alcoholic steatohepatitis by modulating lipid metabolism, fibrosis, and inflammation in rats. Food Funct..

[26] Navik U., Rawat K., Tikoo K. (2022). L-Methionine prevents beta-cell damage by modulating the expression of Arx, MafA and regulation of FOXO1 in type 1 diabetic rats. Acta Histochem..

[27] Joubert M., Jagu B., Montaigne D., Marechal X., Tesse A., Ayer A., Dollet L., Le May C., Toumaniantz G., Manrique A. (2017). The Sodium-Glucose Cotransporter 2 Inhibitor Dapagliflozin Prevents Cardiomyopathy in a Diabetic Lipodystrophic Mouse Model. Diabetes.

[28] Zhang D., Chen Y., Xie X., Liu J., Wang Q., Kong W., Zhu Y. (2012). Homocysteine activates vascular smooth muscle cells by DNA demethylation of platelet-derived growth factor in endothelial cells. J. Mol. Cell. Cardiol..

[29] Deng Y., Li Z., An X., Fan R., Wang Y., Li J., Yang X., Liao J., Xia Y. (2022). Hyperhomocysteinemia Promotes Cardiac Hypertrophy in Hypertension. Oxid. Med. Cell. Longev..

[30] Wu L.L., Wu J.T. (2002). Hyperhomocysteinemia is a risk factor for cancer and a new potential tumor marker. Clin. Chim. Acta.

[31] Hasan T., Arora R., Bansal A.K., Bhattacharya R., Sharma G.S., Singh L.R. (2019). Disturbed homocysteine metabolism is associated with cancer. Exp. Mol. Med..

[32] Plazar N., Jurdana M. (2010). Hyperhomocysteinemia and the role of B vitamins in cancer. Radiol. Oncol..

[33] Curigliano G., Cardinale D., Dent S., Criscitiello C., Aseyev O., Lenihan D., Cipolla C.M. (2016). Cardiotoxicity of anticancer treatments: Epidemiology, detection, and management. CA Cancer J. Clin..

[34] Herrmann J. (2020). Adverse cardiac effects of cancer therapies: Cardiotoxicity and arrhythmia. Nat. Rev. Cardiol..

[35] Lucock M. (2000). Folic acid: Nutritional biochemistry, molecular biology, and role in disease processes. Mol. Genet. Metab..

[36] Sobczynska-Malefora A., Harrington D.J. (2018). Laboratory assessment of folate (vitamin B(9)) status. J. Clin. Pathol..

[37] Shulpekova Y., Nechaev V., Kardasheva S., Sedova A., Kurbatova A., Bueverova E., Kopylov A., Malsagova K., Dlamini J.C., Ivashkin V. (2021). The Concept of Folic Acid in Health and Disease. Molecules.

[38] Ehrlich M. (2002). DNA methylation in cancer: Too much, but also too little. Oncogene.

[39] Zhang D., Wen X., Wu W., Guo Y., Cui W. (2015). Elevated homocysteine level and folate deficiency associated with increased overall risk of carcinogenesis: Meta-analysis of 83 case-control studies involving 35,758 individuals. PLoS ONE.

[40] Liu Z., Luo H., Zhang L., Huang Y., Liu B., Ma K., Feng J., Xie J., Zheng J., Hu J. (2012). Hyperhomocysteinemia exaggerates adventitial inflammation and angiotensin II-induced abdominal aortic aneurysm in mice. Circ. Res..

[41] Sun W., Pang Y., Liu Z., Sun L., Liu B., Xu M., Dong Y., Feng J., Jiang C., Kong W. (2015). Macrophage inflammasome mediates hyperhomocysteinemia-aggravated abdominal aortic aneurysm. J. Mol. Cell. Cardiol..

[42] Yang A., Sun Y., Mao C., Yang S., Huang M., Deng M., Ding N., Yang X., Zhang M., Jin S. (2017). Folate Protects Hepatocytes of Hyperhomocysteinemia Mice from Apoptosis via Cystic Fibrosis Transmembrane Conductance Regulator (CFTR)-Activated Endoplasmic Reticulum Stress. J. Cell. Biochem..

[43] Zoungas S., McGrath B.P., Branley P., Kerr P.G., Muske C., Wolfe R., Atkins R.C., Nicholls K., Fraenkel M., Hutchison B.G. (2006). Cardiovascular morbidity and mortality in the Atherosclerosis and Folic Acid Supplementation Trial (ASFAST) in chronic renal failure: A multicenter, randomized, controlled trial. J. Am. Coll. Cardiol..

[44] Mann J.F., Sheridan P., McQueen M.J., Held C., Arnold J.M., Fodor G., Yusuf S., Lonn E.M., HOPE-2 investigators (2008). Homocysteine lowering with folic acid and B vitamins in people with chronic kidney disease--results of the renal Hope-2 study. Nephrol. Dial. Transplant. Off. Publ. Eur. Dial. Transpl. Assoc. Eur. Ren. Assoc..

[45] Heinz J., Kropf S., Domrose U., Westphal S., Borucki K., Luley C., Neumann K.H., Dierkes J. (2010). B vitamins and the risk of total mortality and cardiovascular disease in end-stage renal disease: Results of a randomized controlled trial. Circulation.

[46] Jardine M.J., Kang A., Zoungas S., Navaneethan S.D., Ninomiya T., Nigwekar S.U., Gallagher M.P., Cass A., Strippoli G., Perkovic V. (2012). The effect of folic acid based homocysteine lowering on cardiovascular events in people with kidney disease: Systematic review and meta-analysis. BMJ.

[47] Pan Y., Guo L.L., Cai L.L., Zhu X.J., Shu J.L., Liu X.L., Jin H.M. (2012). Homocysteine-lowering therapy does not lead to reduction in cardiovascular outcomes in chronic kidney disease patients: A meta-analysis of randomised, controlled trials. Br. J. Nutr..

[48] Octavia Y., Kararigas G., de Boer M., Chrifi I., Kietadisorn R., Swinnen M., Duimel H., Verheyen F.K., Brandt M.M., Fliegner D. (2017). Folic acid reduces doxorubicin-induced cardiomyopathy by modulating endothelial nitric oxide synthase. J. Cell. Mol. Med..

[49] Shi S., Chen Y., Luo Z., Nie G., Dai Y. (2023). Role of oxidative stress and inflammation-related signaling pathways in doxorubicin-induced cardiomyopathy. Cell Commun. Signal..

[50] Goffart S., von Kleist-Retzow J.C., Wiesner R.J. (2004). Regulation of mitochondrial proliferation in the heart: Power-plant failure contributes to cardiac failure in hypertrophy. Cardiovasc. Res..

[51] Li M., Chen J., Li Y.S., Feng Y.B., Gu X., Shi C.Z. (2006). Folic acid reduces adhesion molecules VCAM-1 expession in aortic of rats with hyperhomocysteinemia. Int. J. Cardiol..

[52] Li M., Chen J., Li Y.S., Feng Y.B., Zeng Q.T. (2007). Folic acid reduces chemokine MCP-1 release and expression in rats with hyperhomocystinemia. Cardiovasc. Pathol..

[53] Zhu W., Zhang W., Shou W., Field L.J. (2014). P53 inhibition exacerbates late-stage anthracycline cardiotoxicity. Cardiovasc. Res..

[54] Zhu W., Reuter S., Field L.J. (2019). Targeted expression of cyclin D2 ameliorates late stage anthracycline cardiotoxicity. Cardiovasc. Res..

[55] Liao J., Guo X., Wang M., Dong C., Gao M., Wang H., Kayoumu A., Shen Q., Wang Y., Wang F. (2017). Scavenger Receptor Class B Type 1 Deletion Led to Coronary Atherosclerosis and Ischemic Heart Disease in Low-density Lipoprotein Receptor Knockout Mice on Modified Western-type Diet. J. Atheroscler. Thromb..

[56] Xie Y.P., Lai S., Lin Q.Y., Xie X., Liao J.W., Wang H.X., Tian C., Li H.H. (2018). CDC20 regulates cardiac hypertrophy via targeting LC3-dependent autophagy. Theranostics.

[57] Liao J., An X., Yang X., Lin Q.Y., Liu S., Xie Y., Bai J., Xia Y.L., Li H.H. (2020). Deficiency of LMP10 Attenuates Diet-Induced Atherosclerosis by Inhibiting Macrophage Polarization and Inflammation in Apolipoprotein E Deficient Mice. Front. Cell Dev. Biol..

